# Circadian Rhythms Differ between Sexes and Closely Related Species of *Nasonia* Wasps

**DOI:** 10.1371/journal.pone.0060167

**Published:** 2013-03-26

**Authors:** Rinaldo C. Bertossa, Jeroen van Dijk, Wenwen Diao, David Saunders, Leo W. Beukeboom, Domien G. M. Beersma

**Affiliations:** 1 Chronobiology, Centre for Behaviour and Neurosciences, University of Groningen, Groningen, The Netherlands; 2 Département de Biologie, Université de Sherbrooke, Sherbrooke, Québec, Canada; 3 Evolutionary Genetics, Centre for Ecological and Evolutionary Studies, University of Groningen, Groningen, The Netherlands; 4 University of Edinburgh, Edinburgh, United Kingdom; Simon Fraser University, Canada

## Abstract

Activity rhythms in 24 h light-dark cycles, constant darkness, and constant light conditions were analyzed in four different *Nasonia* species for each sex separately. Besides similarities, clear differences are evident among and within *Nasonia* species as well as between sexes. In all species, activity in a light-dark cycle is concentrated in the photophase, typical for diurnal organisms. Contrary to most diurnal insect species so far studied, *Nasonia* follows Aschoff's rule by displaying long (>24 h) internal rhythms in constant darkness but short (<24 h) in constant light. In constant light, *N. vitripennis* males display robust circadian activity rhythms, whereas females are usually arrhythmic. In contrast to other *Nasonia* species, *N. longicornis* males display anticipatory activity, i.e. activity shortly before light-on in a light-dark cycle. As expected, *N. oneida* shows activity patterns similar to those of *N. giraulti* but with important differences in key circadian parameters. Differences in circadian activity patterns and parameters between species may reflect synchronization of specific life-history traits to environmental conditions. Scheduling mating or dispersion to a specific time of the day could be a strategy to avoid interspecific hybridization in *Nasonia* species that live in sympatry.

## Introduction

Many traits in insects are subject to circadian oscillation. Examples are locomotor activity [1], hatching [2], eclosion [3], emergence from host puparia [4], [5], and mating [6], [7]. Many of these circadian traits are expected to have an adaptive significance [8]. Circadian and annual oscillation of many environmental parameters such as temperature, light and humidity or even predation pressure would cause synchronization of an organism's vital functions with optimal external conditions. Woelfle *et al.* [9] found that cyanobacteria strains with a functioning biological clock defeat strains with non-functioning clocks. Moreover, this advantage is stronger when the internal rhythm is similar to the external light-dark cycle. Considering this potential selective advantage, differences in circadian traits are also expected within species, between sexes or among populations of the same species. For instance, *Drosophila* species from northern and cool latitudes are preferentially active throughout the day whereas species from southern latitudes display a bimodal pattern with activities concentrated at the beginning and end of the photophase. Simunovic and Jaenike [10] found a positive correlation between the midday activity of 11 *Drosophila* species and the latitudinal midpoint of the species range. Furthermore, among species from the same latitudinal region, those that inhabit swampy areas and breed on skunk cabbage have significantly greater midday activity than do mycophagous species, suggesting that both latitude and breeding site influenced the evolution of their daily activity patterns. Other differences in circadian traits may be caused by selective pressure from competing organisms. In fact, exploitation of different temporal niches during the day is a way to avoid competitors. The parasitoids *Eupelmus orientalis* and *E. vuilleti,* for instance, live in sympatry and parasitize the same host. Differences observed in circadian locomotor activity between *Eupelmus* species are thought to reduce competition for the same resources [11].

In more closely related species, temporal isolation may also contribute to species formation. Females of *Drosophila melanogaster* and its sibling species *D. simulans* mate at different times during the day. This allochrony seems to favour reproductive isolation and may have been a cause of speciation in these *Drosophila* species [6]. Also Rivas *et al.* [12] observed small but significant differences in the phase of circadian activity patterns between two species of the sandfly *Lutzomyia*, the main vectors of visceral leishmaniasis in Latin America, which may contribute to their reproductive isolation.

Sex-specific variations in the temporal distribution of specific traits are also present but less studied. Since sexual activities in insects are frequently confined within a specific period of the day, synchronization between sexes is crucial to optimize reproductive success. In females of the turnip moth *Agrotis segetum* the circadian release of sex pheromones at the beginning of the scotophase is endogenously controlled [13] as is pheromone-mediated upwind flight in males during the same hours of the day [14]. Rivas *et al.* [12] found that males of the sandfly *Lutzomyia* initiate their activity a little earlier and have a broader activity peak than females, which tend to be more nocturnal than males. The reason for this difference is not yet known.

Species complexes displaying variations in circadian rhythms are particularly attractive for studying adaptation and the role played by clock genes herein. The parasitic wasp *Nasonia vitripennis* has been extensively used to study photoperiodism [15]–[17]. Recently, the genomes of four *Nasonia* species (*N. vitripennis*, *N. giraulti*, *N. longicornis*, *N. oneida*) were sequenced and annotated [18]. With this resource and the development of more advanced genetic tools like RNAi, *Nasonia* is attracting more interest for addressing evolutionary questions, like the genetic regulation of photoperiodism, which is more difficult in classic model organisms such as *Drosophila melanogaster* [19]. Circadian rhythms in activity and emergence but not eclosion were previously described for *N. vitripennis* males [5]. Here we compare circadian activity rhythms for all four *Nasonia* species under 24 h light-dark cycle, constant darkness and constant light conditions. We interpret the results in the context of what is known about the natural ecology of the species.

## Materials and Methods

### Nasonia strains

*Nasonia* are parasitic wasps of the family Pteromalidae (Hymenoptera) and members of the superfamily Chalcidoidea. *N. vitripennis* is cosmopolitan, but the other three species, *N. giraulti*, *N. longicornis* and *N. oneida*, are endemic to North America [20], [22]. *N. longicornis* occurs in northwestern North America; *N. giraulti* and *N. oneida* are sympatric and occur in eastern North America [20]. *Nasonia vitripennis* split first from the other species lineage between 0.2 and 1 MYA [21] and later, about 0.4–0.5 MYA, *N. giraulti, N. longicornis*, and *N. oneida* diverged from each other [20]. All species parasitize pupae of different fly species found primarily in bird nests and carcasses [22]. *N. vitripennis* is a more generalist and parasitizes Sarcophagidae, Muscidae and Calliphoridae species. The other *Nasonia* species parasitize primarily the calliphorid genus *Protocalliphora* (bird blowflies). The following *Nasonia* strains were used (when known, place and year of collection are indicated in parentheses): *Nasonia giraulti* RV2x(u) (Virginia, USA, 1987), NGDS (eastern North America), VA2TET (Virginia, USA, 2007, Tetracycline treated), PA233F (Pennsylvania, USA, 1989), VA1TET (Virginia, USA, 2007, Tetracycline treated); *Nasonia longicornis* IV7 (Utah, USA), IDB418(u) (Idaho, USA), MN8510 (Minnesota, USA), UTB316.16 (Utah, USA, 1990); *N. oneida* NY1136 (NY, USA, 2005); *Nasonia vitripennis* AsymC (Leiden, NL, 1971, cured from Wolbachia bacteria), HV3 (Hoge Veluwe, NL, 2006), Ita2 (Piedmont, Italy, 2006), LabII (Leiden, NL), Sal29 (New York, USA, 2007). Collection of *Nasonia* strains enjoyed permission of local authorities and did not involve endangered or protected species. Strain information comes from Darling and Werren [22], van den Assem and Jachman [23], Grillenberger et al. [24] and Raychoudhury [20]. Once in the lab, *Nasonia* strains are reared according to standard techniques [5].

### Recording and analyses of activity rhythms

Males and females were collected at the black pupal stage and allowed to further develop in same sex groups of 10 to 15 individuals in 63×11 mm polystyrene tubes at 20°C in an 18 h∶6 h light-dark cycle. Once eclosed, virgin wasps were used immediately for recording individual activity rhythms as described in Bertossa *et al.* [5]. Males and females of each species were analyzed simultaneously. All animals were subjected to a protocol comprising an initial entraining phase of at least 4 full days in a 16 h∶8 h light-dark cycle (LD) followed by constant darkness (DD) for 8 days and subsequent 8 days in constant light (LL). Light intensity was approximately 30 lx. Activity data were averaged over 10 minute interval-bins and analyzed with ChronOSX v2.3.2 [25]. For average activity plots in LD, median, 25-, and 75-percentiles were calculated on the last three days of the LD entraining phase over all wasps. For the same period of time, the Centre point of Gravity (CoG, i.e. the activity peak in a fitted 24 h sine wave) was calculated in ChronOSX for each wasp.

### Measuring Tau in DD and LL

The circadian period of the activity rhythm (tau, τ) in constant conditions was calculated for each wasp with ChronOSX according to the periodogram analysis by Sokolove and Bushell [26]. The first day – in DD or LL – was omitted from the calculation in order to exclude influences from the preceding light condition [5]. Double plots were first created in ChronOSX and monitored by eye to exclude dead wasps and individuals whose rhythmicity could not be clearly assessed due to e.g. presence of multiple rhythms (see figure S1). However, in order to use an unbiased method in the determination of arrhythmic animals, wasps whose ratio between the main peak value of the Qp periodogram statistics and the Qp value corresponding to the 0.01 confidence threshold (simply called “ratio-to-p”) was below 5.5 were considered arrhythmic. For examples of activity plots with increasing ratio-to-p values see figure S2.

### Statistical analysis

All statistical analyses were performed in R, version 2.13.0 [27]. Differences among groups (e.g. response variables τ and CoG) were analyzed with mixed-effects models in which explanatory variables (fixed factors) were species, strains, experiments, light condition, and sex. “Strains (or species) within experiments” was fed into the model as random factor. Full models (i.e. with all factors and interactions) were simplified by removing non-significant explanatory factors. Best fit was assessed by comparing likelihoods of complex and simplified model with a Chi-squared test. Response variables were then tested for significant factors with post-hoc tests (Tukey HSD).

## Results

Activity rhythms were analyzed under different light settings between sexes in all four known *Nasonia* species. These included the standard laboratory strain of each species (Werren et al 2010) and individuals of additional strains for each species (see table 1). Standard strains were: RV2X(u) for *N. giraulti*, IV7 for *N. longicornis*, NY1136 for *N. oneida*, and AsymC for *N. vitripennis*. Wasps were initially entrained for at least four days in LD 16∶8 h. Two phases in constant conditions (8 days in DD and 8 days in LL) followed directly after the LD phase. We present the analysis for the standard strains first and comparisons within species later.

**Table 1:** τ and ‘ratio-to-p’ values for all *Nasonia* strains in constant darkness (DD).

CONST CONSTANT DARKNESS ANT DARKNESS															
		Females							Males						
Species	Line	Tau (τ)		SEM	ratio to p	% rhythmic	% non-rhythmic	Total wasps	Tau (τ)		SEM	ratio to p	% rhythmic	% non-rhythmic	Total wasps
***N. giraulti***	RV2X(u)	23.7		0.11	14.2	95	5	37	25.0		0.31	8.1	68	32	31
	NGDS	23.2		0.27	13.4	100	0	6	23.5		1.14	8.7	83	17	6
	VA2TET	23.5		0.20	19.1	100	0	6	24.3		0.38	10.5	100	0	5
	PA233F	23.7		0.24	16.0	100	0	6	24.4		0.62	11.0	100	0	6
	VA1TET	22.5		0.40	13.6	100	0	5	23.6		0.65	6.4	33	67	6
	**Means:**	**23.5**	**[23.3]**	**0.10**	**14.7**	**97**	**3**	**60**	**24.6**	**[24.2]**	**0.25**	**8.8**	**72**	**28**	**54**
***N. longicornis***	IV7	25.0		0.14	9.1	100	0	18	26.0		0.46	7.6	72	28	18
	IDB418(u)	24.2		0.22	13.2	100	0	6	27.6		0.46	11.7	100	0	6
	MN8510	24.0		0.09	15.7	100	0	6	24.5		0.21	13.7	100	0	6
	UTB316.16	26.0		0.16	12.8	100	0	6	27.1		0.51	7.7	83	17	6
	**Means:**	**24.9**	**[24.8]**	**0.14**	**11.5**	**100**	**0**	**36**	**26.2**	**[26.3]**	**0.30**	**9.6**	**83**	**17**	**36**
***N. oneida***	NY1136	24.6		0.12	14.6	100	0	19	26.0		0.20	12.6	94	6	18
***N. vitripennis***	AsymC	26.4		0.08	10.1	78	22	41	25.7		0.06	16.5	98	3	40
	HV3	24.5		0.26	11.2	100	0	6	24.4		0.39	10.5	100	0	6
	Ita2	25.4		0.24	11.8	83	17	6	25.5		0.16	12.8	100	0	5
	LabII	26.2		0.23	8.9	80	20	5	26.0		0.11	18.5	100	0	6
	Sal29	25.2		0.29	9.7	100	0	6	25.8		0.30	12.6	80	20	5
	**Means:**	**25.9**	**[25.5]**	**0.12**	**10.3**	**83**	**17**	**64**	**25.6**	**[25.5]**	**0.08**	**15.5**	**97**	**3**	**62**

Tau (τ, in hours), ratio-to-p, standard error (SEM), and number of wasps used are indicated for each strain. Species means are indicated in bold (except for column ‘Total wasps’, which indicates the total amount of wasps per each species). Tau means are weighted and, respectively, non-weighted (in brackets). For significant differences see figures 4 and 5.

### Standard *Nasonia* strains

#### Nasonia giraulti

**Figure 1:**
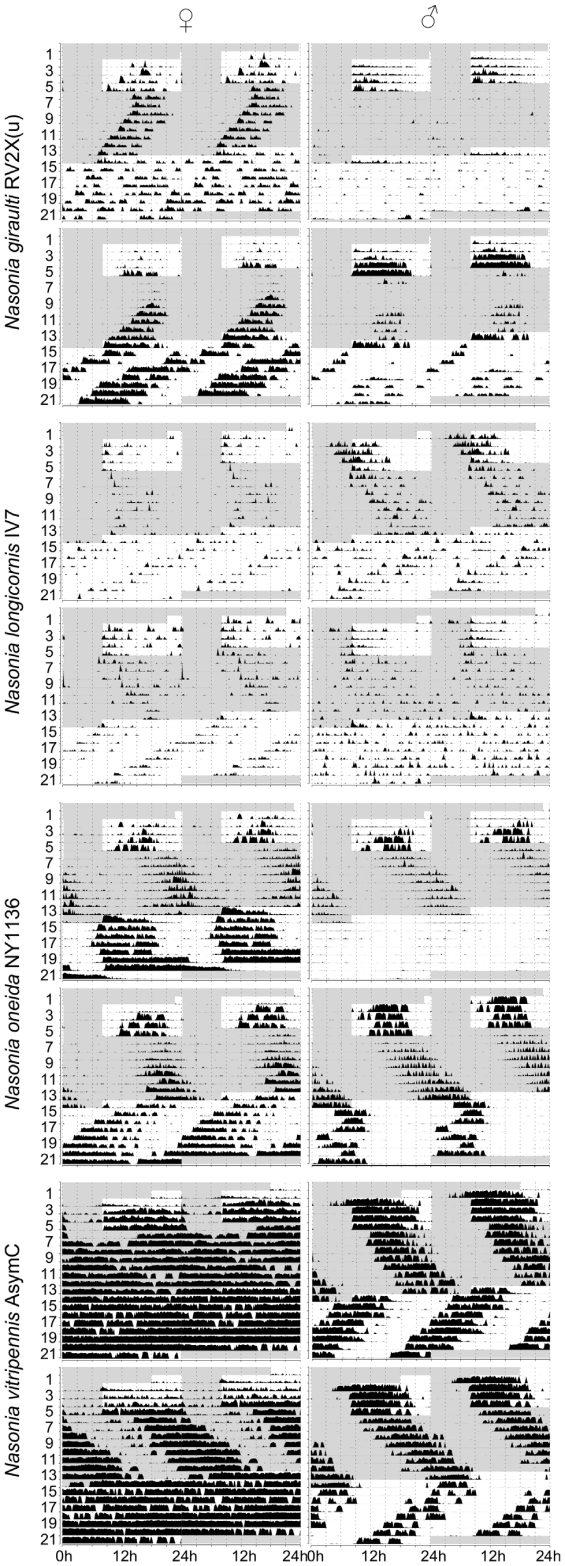
Typical actograms for different *Nasonia* species. Representative actograms (double plotted) of male and female *Nasonia* are shown for standard strains in each species. Wasps were first entrained in LD 16∶8 (light phase from 8:00 to 24:00) for at least 4 days and then exposed to constant darkness (indicated with a gray background) for 8 days followed by constant light (white background). Y- and x-axes indicate time in days and hours, respectively.

**Figure 2:**
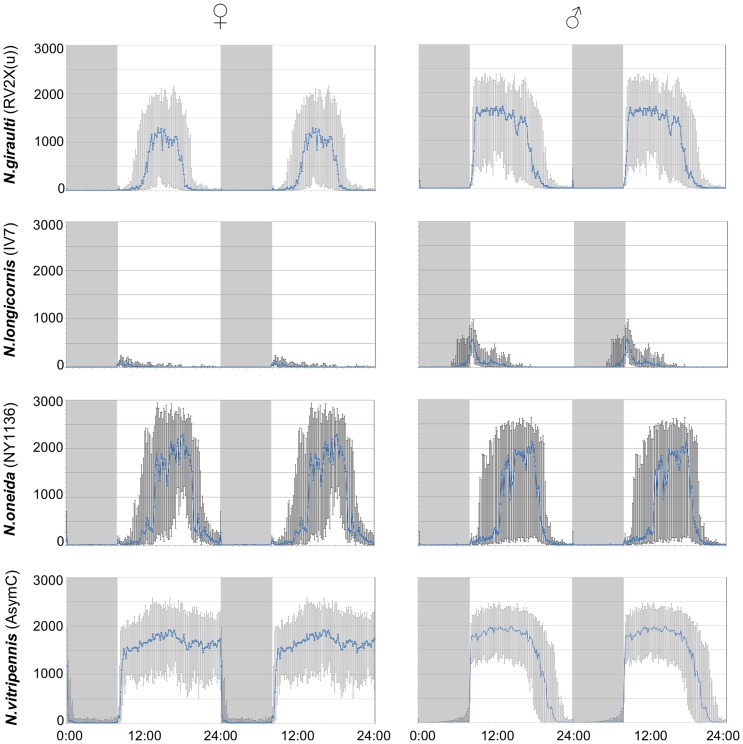
Average activity during LD in standard strains. The median (with lower and upper quartiles) of the activity over the last three days in LD (double plotted) is indicated for each sex in the standard strains used for each species. Gray background indicates darkness. Y-axis: activity (pixel changes/minute); x-axis: hour of the day.

**Figure 3:**
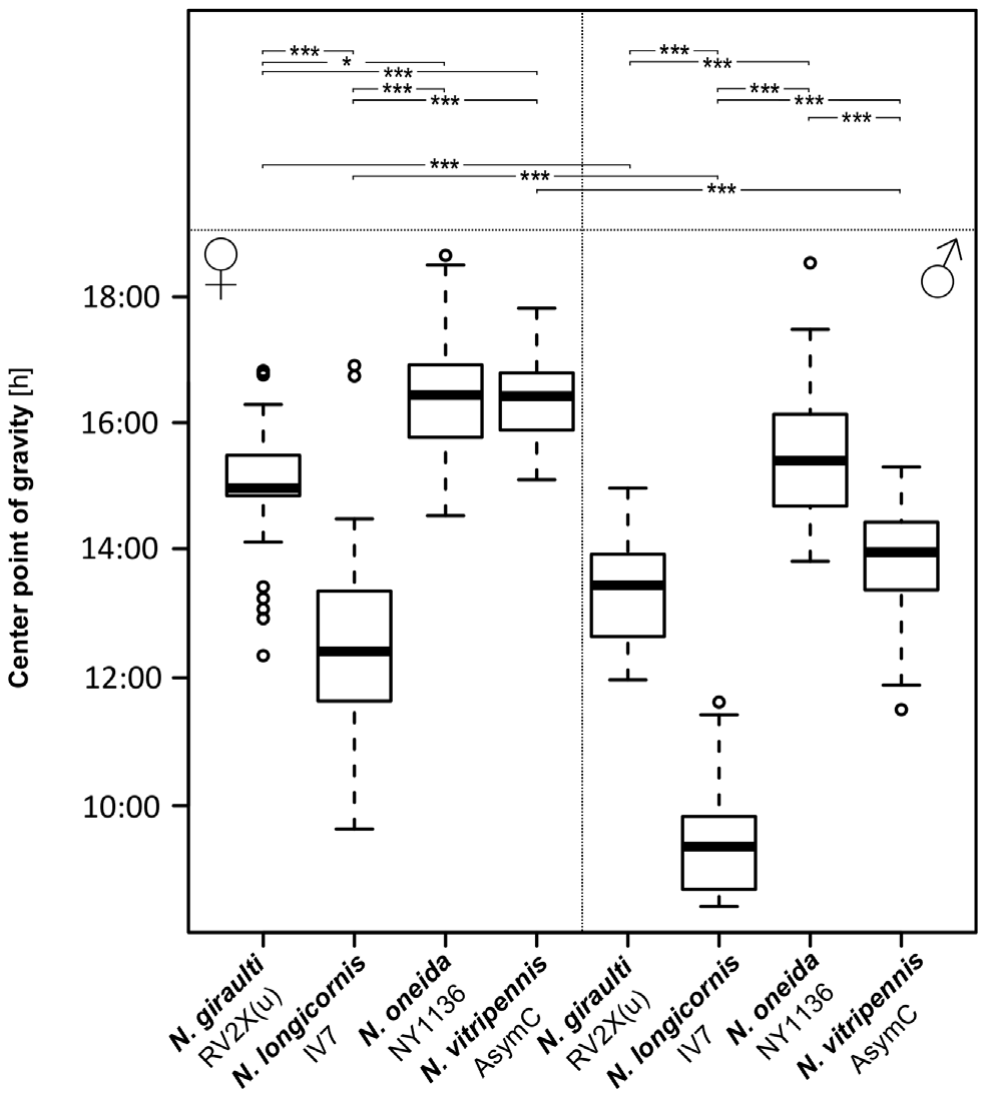
Centre point of gravity of main *Nasonia* strains. Box-plots summarize Centre-point-of-gravity (CoG) values in LD for the standard strains used in each species. Significant differences are indicated with asterisks in the top panel: among strains (within one sex) on top, and between sexes (within each strain) at the bottom (* * = p*<0.05; ** * = p*<0.01; *** * = p*<0.001). Circles in box plots represent outliers. A summary of the data is given in table 3.

**Figure 4:**
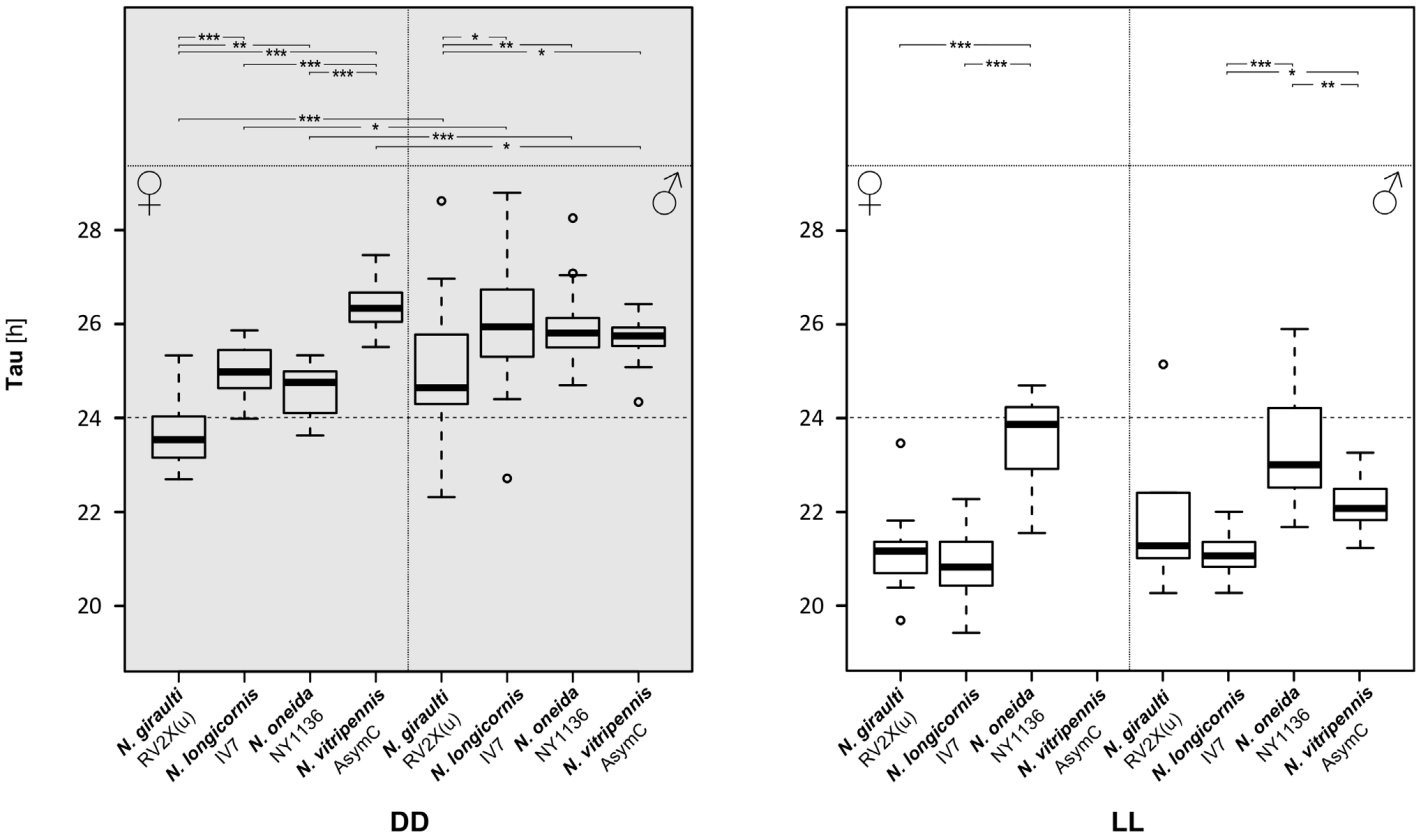
Τ of standard *Nasonia* strains. Box-plots summarize τ values in constant darkness (DD) and constant light (LL) for the standard strain of each species. Significant differences are indicated with asterisks in the top panel: among strains (within one sex) on top, and between sexes (within each strain) at the bottom (* * = p*<0.05; ** * = p*<0.01; *** * = p*<0.001). Circles in box plots represent outliers. Differences between τ values in darkness and light are always significant. A summary of the data is given in tables 1 and 2.

*Nasonia giraulti* RV2X(u) displays the greatest variation among individuals of all four species. Activity patterns can vary from short bouts of weak to sustained activity to longer bouts of intense activity (fig. 1). In males, activity begins after light-on and decreases after 9–12 h. In both constant DD and LL, on the contrary, activity is just a fraction of that of LD and irregular, with few and scattered activity bouts. An increase in activity is seen in some males at the beginning of LL however, after this, usually activity becomes again irregular as in DD. Indeed, rhythmicity is not strong in RV2X(u) males, both in DD (ratio-to-p is 8.1) and in LL (9.8). Around 67% of males are rhythmic in DD, whereas the majority (77%) is arrhythmic in LL (tables 1 and 2). Females have usually more pronounced activity patterns than males. In LD their activity is concentrated in the middle of the day (fig. 1 and 2). The peak of activity (Centre point of Gravity, CoG) can vary considerably, but this is observed in all species (fig. 3, table 3). τ of females in DD is the only one of all standard strains that is lower than 24 h (23.7 h, fig. 4). In LL, although relatively more females than males are rhythmic, the majority is non-rhythmic (57%, table 2). Their rhythmicity, however, is more evident than in males (ratio-to-p is 14.2 in DD and 10.7 in LL, tables 1 and 2).

**Table 2:** τ and ‘ratio-to-p’ values for all *Nasonia* strains in constant light (LL).

CONSTANT LIGHT															
		Females							Males						
Species	Line	Tau (τ)		SEM	ratio to p	% rhythmic	% non- rhythmic	Total wasps	Tau (τ)		SEM	ratio to p	% rhythmic	% non-rhythmic	Total wasps
***N. giraulti***	RV2X(u)	21.1		0.20	10.7	43	49	37	22.0		0.85	9.8	33	67	15
	NGDS	21.0		0.39	14.0	100	0	6	20.0		1.04	10.1	100	0	2
	VA2TET	20.9		0.55	12.4	100	0	6	21.0		–	11.0	50	50	2
	PA233F	21.3		0.51	16.6	100	0	6	–		–	–	0	100	3
	VA1TET	19.4		0.60	16.2	80	20	5	21.3		0.54	8.6	75	25	4
	**Means:**	**20.9**	**[20.8]**	**0.18**	**13.0**	**63**	**32**	**60**	**21.4**	**[21.1]**	**0.47**	**9.6**	**42**	**58**	**26**
***N. longicornis***	IV7	20.8		0.18	11.7	100	0	18	21.1		0.16	8.8	69	31	16
	IDB418(u)	20.3		0.14	16.4	100	0	6	20.6		0.22	14.3	100	0	6
	MN8510	21.4		0.15	15.7	100	0	6	21.9		0.30	9.3	83	17	6
	UTB316.16	22.2		0.25	17.2	100	0	6	22.1		0.48	11.7	67	33	6
	**Means:**	**21.1**	**[21.2]**	**0.14**	**14.0**	**100**	**0**	**36**	**21.3**	**[21.4]**	**0.15**	**10.6**	**76**	**24**	**34**
***N. oneida***	NY1136	23.5		0.29	12.6	87	13	15	23.4		0.48	8.9	89	11	9
***N. vitripennis***	AsymC	–		–	–	0	100	36	22.1		0.08	16.2	97	3	39
	HV3	–		–	–	0	100	6	21.1		0.25	13.2	100	0	5
	Ita2	21.9		0.14	12.1	60	40	5	21.4		0.59	11.2	100	0	5
	LabII	–		–	–	0	100	3	22.7		0.31	16.2	100	0	6
	Sal29	23.1		0.46	6.9	67	33	6	21.8		0.15	15.2	100	0	5
	**Means:**	**22.6**	**[22.5]**	**0.35**	**9.1**	**13**	**88**	**56**	**22.0**	**[21.9]**	**0.09**	**15.4**	**98**	**2**	**60**

**Figure 5:**
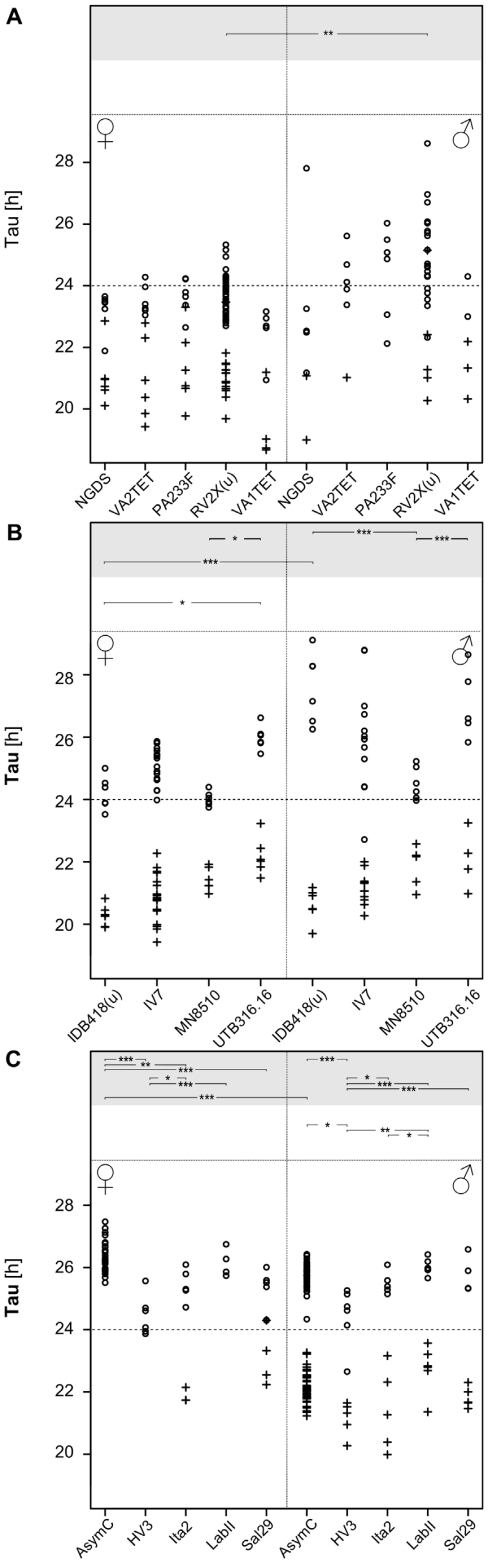
Τ in all tested strains for each *Nasonia* species. τ values are indicated for all strains used in *N. giraulti* (**A**), *N. longicornis* (**B**), and *N. vitripennis* (**C**). Circles (o) and crosses (+) are used to distinguish τ values in DD an LL, respectively. Significant differences (among strains within each sex, and between sexes within each strain) are indicated in the top panels with asterisks (* * = p*<0.05; ** * = p*<0.01; *** * = p*<0.001). Background is gray for DD and white for LL, respectively. Differences in τ between constant darkness and constant light are always significant. A summary of the data is given in tables 1 and 2.

Tau (τ, in hours), ratio-to-p, standard error (SEM), and number of wasps used are indicated for each strain. Species means are indicated in bold (except for column ‘Total wasps’, which indicates the total amount of wasps per each species). Tau means are weighted and, respectively, non-weighted (in brackets). For significant differences see figures 4 and 5.

**Table 3:** CoG values for all *Nasonia* strains.

		Females				Males			
Species	Line	CoG		SEM	Total wasps	CoG		SEM	Total wasps
***N. giraulti***	RV2X(u)	15:00		0:10	37	13:20		0:08	31
	NGDS	14:06		0:24	6	12:16		0:20	6
	VA2TET	15:34		0:31	6	13:22		0:16	6
	PA233F	14:43		0:17	6	13:00		0:31	6
	VA1TET	14:15		0:21	5	13:15		0:26	6
	**Means:**	**14:53**	**[14:44]**	**0:08**	**60**	**13:10**	**[13:02]**	**0:07**	**55**
***N. longicornis***	IV7	12:39		0:27	18	9:31		0:13	18
	IDB418(u)	12:20		0:13	6	12:18		0:38	6
	MN8510	13:30		0:56	6	11:19		0:20	6
	UTB316.16	12:46		1:04	6	12:52		0:32	6
	**Means:**	**12:45**	**[12:49]**	**0:19**	**36**	**10:50**	**[11:30]**	**0:17**	**36**
***N. oneida***	NY1136	16:24		0:14	19	15:33		0:16	18
***N. vitripennis***	AsymC	16:24		0:06	41	13:58		0:10	40
	HV3	15:23		0:38	6	11:58		0:25	6
	Ita2	12:41		0:29	6	12:00		0:18	6
	LabII	15:41		0:22	5	13:10		0:15	6
	Sal29	11:32		0:25	6	11:01		0:14	5
	**Means:**	**15:27**	**[14:20]**	**0:14**	**64**	**13:17**	**[12:25]**	**0:10**	**63**

**Figure 6:**
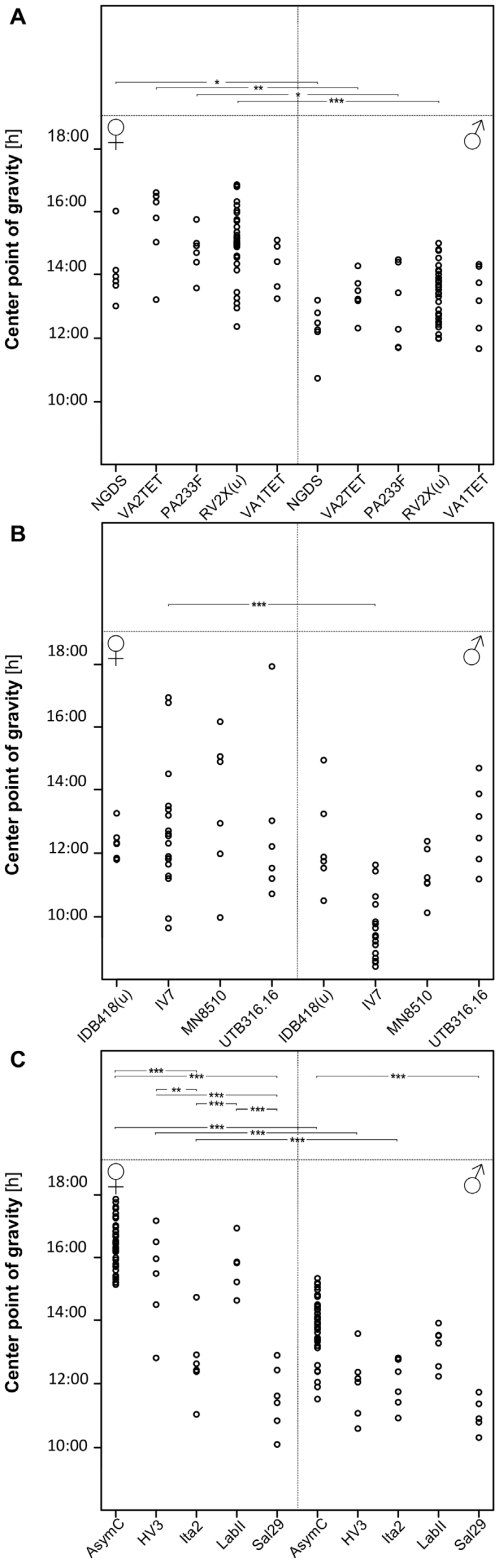
CoG for all tested strains of four Nasonia species. CoG values are indicated for all strains used in *N. giraulti* (**A**), *N. longicornis* (**B**), and *N. vitripennis* (**C**). Significant differences are indicated in the top panel with asterisks: among strains (within each sex) on top, and between sexes (within each strain) at the bottom (* * = p*<0.05; ** * = p*<0.01; *** * = p*<0.001).A summary of the data is given in table 3.

CoG (hours:minutes; light phase is from 8:00 to 24:00), standard error (SEM), and number of wasps used are indicated for each strain. Species means are indicated in bold (except for columns ‘Total wasps’, which indicate the total amount of wasps per each species). CoG means are weighted and, respectively, non-weighted (in brackets). For significant differences see figures 3 and 6.

#### Nasonia longicornis

*N. longicornis* IV7 has quite unique features compared with the other standard strains. It is the strain with the lowest levels of activity, both in males and females (fig. 2). Further, in LD both males and females have the earliest activity peak compared with other strains: CoG of males is already 1.5 hours after light-on (9∶30 h) whereas female activity peaks at 12∶40 h (fig. 3 and table 3). Notably, *N. longicornis* is the only species in which males show anticipatory activity before light-on in LD (fig. 1 and 2). Males and females have similar patterns of activity, concentrated in short and often scattered bouts if compared to the more prolonged bouts of *N. giraulti* or the even longer bouts of *N. vitripennis* (fig. 1). Although all females were found to be rhythmic in DD their rhythmicity is not very pronounced (ratio-to-p is 9.1). Rhythmicity of *N. longicornis* males is less pronounced than that of females (around 70% of males are rhythmic both in DD and LL, table 1 and 2) and is the least strong of all standard strains analyzed (ratio-to-p is 7.6 in DD and 8.8 in LL).

#### Nasonia oneida

Only one strain of *N. oneida* was analyzed (NY1136). Not surprisingly it has many features in common with *N. giraulti*, from which it is assumed to have recently split [20]. For instance, activity patterns – with variably sized bouts of intense activity – resemble those of *N. giraulti* (fig. 1) and females have activity concentrated in the middle of the light phase in LD (fig. 1 and 2). However, contrary to *giraulti*, also *oneida* males have activity concentrated in the middle of the day (fig. 1 and 2) and, in LD, activities peak also at different times than observed in *giraulti* (fig. 3 and table 3). Under constant conditions, most male and female *N. oneida* are rhythmic in both DD and LL and, in LL, have the largest τ of all standard strains (23.5 h for females and 23.4 h for males, fig. 4 and table 2). Rhythms are usually readily apparent in both sexes and the three light conditions.

#### Nasonia vitripennis

In *N. vitripennis* wasps of strain AsymC both females and males display intense and sustained activity in the light phase of LD and, typically, also in constant conditions (fig. 1 and 2). In LD, males begin activity immediately after light-on and cease activity after about 10–13 h (fig. 1 and 2), although some individuals may continue for the whole 16 h of light (not shown). After light-on in LD, females do not usually start activity as fast as males but are active throughout the light phase (fig. 1 and 2). Furthermore, females appear the only ones to consistently display a residual activity during the dark phase in LD (fig. 1 and 2). In LD, activity peaks at 16∶24 h in females and 13∶58 h in males (fig. 3 and table 3). In constant conditions, activity bouts are frequently very sharp, with low to absent background activity. In DD, τ is 25.7 h in males and 26.4 h in females – the largest of all female groups in DD (fig. 4; table 1) and more males (98%) than females (78%) are rhythmic. In LL, males maintain rhythmicity albeit with a τ of 22.15 h while females are arrhythmic (table 2).

### Additional strains

#### Nasonia giraulti

**Figure 7:**
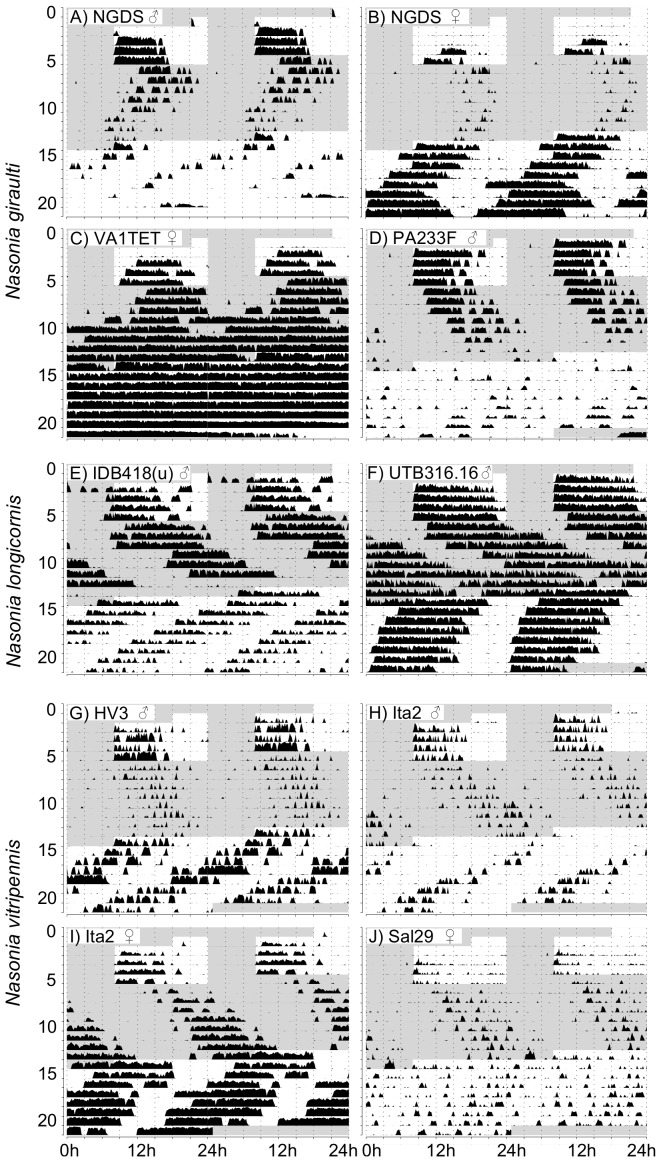
Actograms of additional *Nasonia* strains. *N. giraulti*: NGDS males can show strong activity at the beginning (A); conspicuous LL activity is seen in some NGDS females (B); an example of VA1TET female with strong activity pattern (C); a male PA233F starting activity at light-on in LD (D). *N. longicornis*: IDB418(u) males can have robust and sustained activity similar to that observed in AsymC males (E, anticipation in LD is evident); an example of a UTB316.16 male showing strong activity but no anticipation in LD (F). *N. vitripennis*: in some *vitripennis* strains males can be less active in DD than are AsymC males (G and H) and females of some *vitripennis* strains can be rhythmic in LL (I and J).

Four additional *N. giraulti* strains were analysed. No significant differences among strains were found in τ (fig. 5A), CoG (fig. 6A), or activity patterns in LD (fig. S3). However, sexes (within the same strain) differ in their LD activity peaks in four out of five strains (fig. 6A). In contrast, for τ in constant DD no differences between sexes were observed except in the standard strain RV2X(u) (fig. 5A). Note however the large variation observed within certain strains. For instance, for the standard strain, τ of male wasps in DD varied between 22.3 and 28.6 h. Females, instead, show less variation of τ in DD, are mostly rhythmic and their rhythms are quite pronounced, with ratio-to-p ranging from 13.4 to 19.1 (table 1). Also in LL, females of most *N. giraulti* strains are rhythmic with well supported rhythms while fewer males are rhythmic in LL (although a high mortality among males reduced available samples, table 2). CoG of *N. giraulti* females is at around 14∶50 h; that of males at 13∶10 h. Representative double-plots of *N. giraulti* strains are presented in figure 7. In general, activity patterns resemble those of main strain RV2X(u).

#### Nasonia longicornis

Although within single *N. longicornis* strains τ varies less than within *N. giraulti* strains, greater differences are observed among *longicornis* strains used here and some are significant (fig. 5B). In these strains τ in DD is generally greater than in *giraulti* strains, both in males (26.2 h) and in females (24.9 h). In LL however, τ are similar to those observed in *giraulti* strains (table 2 and fig. 5). Similar to *N. giraulti*, *N. longicornis* strains show usually more evident rhythms in females than in males, in both light conditions (tables 1 and 2). For instance, the average ratio-to-p in DD is above 11 for females while that of males is below 10 (table 1). This difference is even more accentuated in LL (table 2). Female LD activity peaks roughly 2 h earlier than in *N. giraulti* (12∶49 h) while that of males is maximal at around 11∶30 h, with exception of strain IV7 which peaks earlier (9∶31 h, table 3 and fig. 6B). Activity patterns of the additional *N. longicornis* strains are quite similar to those of the standard IV7 strain. However, some important differences can be noted. For instance, males in some strains can have robust and sustained activity similar to that observed in AsymC (e.g. fig. 7E and F, and S3) and have frequently also a pronounced rhythmicity (tables 1 and 2). Even if lower than compared to other species, LD activity in females of *N. longicornis* strains is not as low as observed for IV7 females (compare fig. 2 with S3). Anticipation is seen in most males of *N. longicornis* strains (fig. 7 and S3). Other strains have an activity pattern similar to that of IV7 (not shown).

#### Nasonia vitripennis

Most significant differences are observed among *N. vitripennis* strains (fig. 5C, 6C and S3). However, differences between sexes are significant only for CoG but not τ (except for AsymC). In general, in DD females display a τ greater than observed in other species (table 1). Furthermore, in most strains females are clearly non-rhythmic in LL with the exception of Ita2 and Sal29 (table 2). Males are usually highly rhythmic in both DD and LL. Activity patterns of HV3 are similar to those of AsymC. Despite having a strong supported rhythmicity (table 1), HV3 and Ita2 males are less active in DD than AsymC males (fig. 7, G and H). Ita2 females can be rhythmic in LL (fig. 7I). Sal29 females show rhythmicity in LL but this is not strongly supported (fig. 7J). AsymC and LabII activity patterns are very similar. Continuous activity throughout the light phase does not seem to be the rule in *N. vitripennis* females (fig. S3). Additionally, night activity is only present in laboratory lines (AsymC and LabII).

### Strains pooling and comparison among species

**Figure 8:**
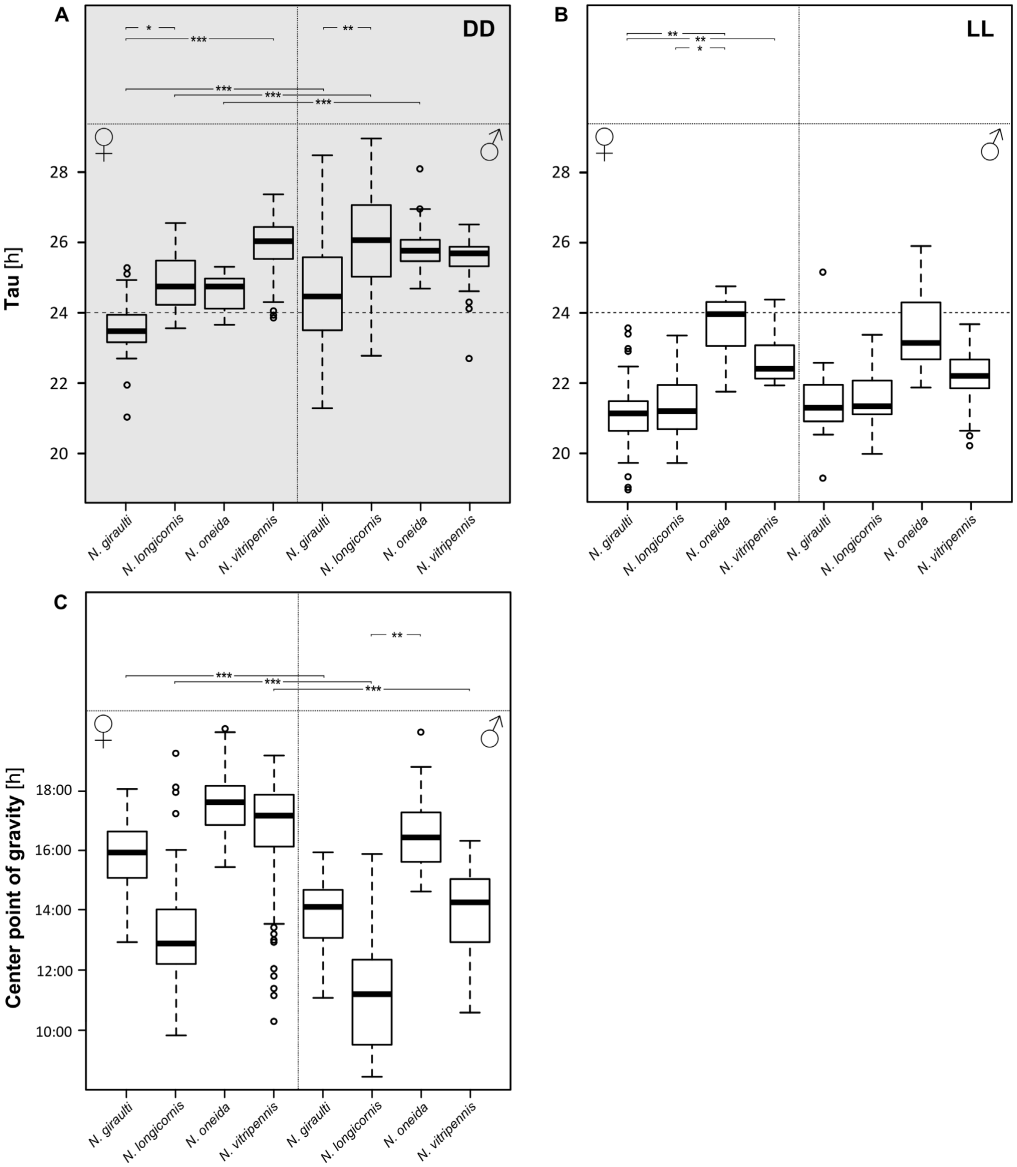
τ and CoG differences among *Nasonia* species. Data from distinct strains within one species were pooled in order to find differences among *Nasonia* species for τ and CoG. Significant differences are indicated with asterisks in the top panel: among strains (within one sex) on top, and between sexes (within each strain) at the bottom (* * = p*<0.05; ** * = p*<0.01; *** * = p*<0.001).Circles in box plots represent outliers. Differences are based on a model that accounts for different amount of data per strain. Despite pooling, significant differences are found among species and between sexes. Differences between τ values in darkness and light are always significant.

Data of all strains within a species were pooled in order to assess differences among species. (fig. 8). Difference of τ between DD and LL is significant for all species (mixed-effects model, effect of light: *χ* ^2^ = 726.15, *p*<2.2e-16). Furthermore, in DD but not in LL, females have a significantly lower τ compared to males (except *N. vitripennis*). In females τ differs significantly also between *N. giraulti* and two other species: *N. longicornis* and *N. vitripennis* in DD, and *N. oneida* and *N. vitripennis* in LL. As for CoG, differences between sexes are in most species significant (except *N. oneida*).

Circadian activity rhythms in *Nasonia* species display some characteristic hallmarks and can be formally classified by general features as well as elements typical of LD and constant light conditions. General features are: type of activity pattern (i.e. length and intensity of activity bouts), activity patterns at phase changes, and regular pattern vs. presence of abrupt changes (over days or conditions). Elements specific for LD are: Centre point of Gravity, activity distribution over the light phase, onset of activity (i.e. at light-on or delayed), presence of background activity in the dark phase, and anticipation. Finally, activity in constant conditions can be rhythmic (i.e. meaningful τ) or not. With these elements in mind, activity patterns of *Nasonia* species have been classified in table S1. Representative double plots for *Nasonia* species are shown in Figure S4.

## Discussion

*Nasonia* species offer a unique opportunity for studying species formation because, under special circumstances, they can be readily crossed [18]. Furthermore, species exhibit consistent differences in important life history traits, such as host preference [28], learning and memory [29], mating or aggressive behaviour [20], [30]–[32]. Circadian rhythms are a class of traits which also show species-specific differences in insects and have been clearly associated with species formation [33]. Variations in endogenous clock genetics and connected phenotypic outputs – such as circadian rhythms – deserve therefore particular attention for understanding how populations differentiate and form new species. We have previously shown that *Nasonia vitripennis* wasps display robust circadian rhythms [5]. Here we have reported on activity rhythms of both sexes of several strains of *Nasonia* wasps. The protocol used comprised an entraining phase of at least four days in LD 16:8 h followed by eight days in DD and completed by eight days in LL. A longer protocol was not possible due to the restricted lifespan of the wasps. We chose not to provide additional food as it would disturb their circadian rhythms and the automatic registration. A different protocol could be recording activity in DD and LL separately, both after an entraining period in LD, as done by Stelzer et al. in a study on bumblebees [34]. Another important restriction in our experiments was the use of virgin wasps in order to standardize settings. Circadian rhythms in insects may be also influenced by mating status [35], as well as several other factors, like temperature and social environment [36].

### *Nasonia* wasps obey Aschoff's rule

An initial observation is that in all *Nasonia* species τ in DD is always significantly greater than in LL. Furthermore, with the exception of *N. giraulti* females, τ in DD is larger than 24 h, while in LL it is always smaller than 24 h (fig. 4, 5 and 8). According to Aschoff's rule, this situation is characteristic for diurnal animals [37] and, besides the bumblebee, which has a τ around 24 h in DD and shorter than 24 h in LL [34], *Nasonia* is the only diurnal insect so far which strictly follows Aschoff's rule [38]. τ in the blow fly *Calliphora vicina*, for instance, lengthens upon transfer from DD to LL when light intensity is below 2 lx while more and more flies become arrhythmic above 2 lx [39]. The same is true even in the honey bee *A. mellifera* which is, as *Nasonia*, a hymenopteran [40]. Although several strains of *Nasonia* are rhythmic under approximately 30 lx, a shift in τ under different light intensities cannot be ruled out as the same light intensity was used throughout all experiments.

### Similarities and differences among *Nasonia* species and sexes

A τ smaller than 24 h in DD could suggest nocturnal-type behaviour in *N. giraulti* females as does a τ near 24 h in LL in *N. oneida* females. However, double-plots of both *N. giraulti* and *N. oneida* in LD do not indicate nocturnal activity in females of these species. The fact that *N. giraulti* wasps mate within the host puparium [30], [31], before emergence, a place somewhat protected against direct external light, may make them less affected by light fluctuations. Bertossa *et al.* [5] found that, in LD 16∶8, emergence of *N. vitripennis* AsymC virgin males from their host puparium is rhythmic and happens around light-on while eclosion is arrhythmic. Assuming that the latter extends to all *Nasonia* species, one could speculate that mating in *N. giraulti*, if correlated with an arrhythmic eclosion, would be arrhythmic too, i.e. not influenced by the LD cycle, despite adult activity being rhythmic. Conversely, mating in *N. vitripennis*, which occurs after emergence from the host puparium [30], [31], is expected to follow a circadian rhythm. Another interpretation could be derived from Pittendrigh's hypothesis: a circadian clock tracking dawn would have a τ larger than 24 h in DD, while it would be shorter than 24 h if it would track dusk [41]. If this could underlie the differences in τ between *N. vitripennis* and *N. giraulti* females in DD (fig. 4 and 8) which traits would be affected? More specific experiments, aimed at testing associations between activity rhythms and important life-history traits, are needed to answer this question.

Clear differences in male anticipation in LD are apparent among *Nasonia* species (females show virtually no anticipation). *N. longicornis* displays the most pronounced anticipation (and correlated early CoG, table 3), followed by *N. vitripennis*, whereas *N. giraulti* and *N. oneida* have virtually no anticipation (fig. 2 and S3). Anticipation in *N. longicornis* may be a strategy to compete against *N. vitripennis* males with which they live in sympatry. However, *N. longicornis* males do not differ much from *N. vitripennis* males in terms of within-host-mating, dispersion and aggression in contrast to *N. giraulti* males which do disperse [31]. So, whether male anticipation has a particular adaptive significance remains to be determined. In that respect, the difference observed between a strong anticipation in male emergence [5] and a negligible one in activity (fig. 2) shows that circadian rhythms can appear quite different depending on the conditions in which an animal's activity is recorded and the type of behaviour measured: in this case whether the circadian activity is of emerging males vs. simple locomotion (without e.g. presence of hosts). Males inside the host may be influenced by other factors, like presence of other males, particular odours, light coming through the puparium wall, or age.

A clear difference between males and females is the onset of activity at light-on in *N. giraulti*. This happens abruptly in males, whereas a delay is observed in females (fig. 2 and S3). This difference could be correlated either with male dispersion and/or within-host-mating, both present in *N. giraulti* but not in other species [31]. Dispersal may be safer in the middle part of the light phase, while searching for and parasitizing hosts at the place of emergence has to be maximized and can hence be done as soon as and as long as there is light. In that respect it would be highly informative to know whether there are differences in dispersal at different moments in an LD light protocol. Even if in *N. giraulti* mating occurs within the host, a premature onset of activity in females and possible emergence before mating may be prevented by their delayed activity onset. The fact that virtually all AsymC females are arrhythmic in LL and show residual nocturnal activity may have to do with their prolonged exposure to laboratory conditions. In fact, this is not observed in more recent strains obtained from the wild, such as Ita2 and Sal29 (fig. 5 and S3).

### Similarities and differences among strains

Circadian rhythms dynamics are readily affected by adaptation to local climatic conditions, such as latitude, altitude or humidity [10], [42], [43] and may explain differences in τ and CoG observed among *Nasonia* species, as discussed above, but also among strains. A trait related to circadian rhythms in *Nasonia* is photoperiodism, apparent in the form of diapause induction in short days [15], [44], [45]. Whether the circadian system in insects, apparent in overt circadian rhythms, and photoperiodism rely on a unique genetic system comprising canonical clock genes or whether these affect both phenotypes through different pathways is still unclear [19], [46], [47]. While it is undisputed that both systems share components (i.e. clock genes), the magnitude of communality also depends on the insect system being considered, indicating that the genetic architecture(s) underlying circadian and seasonal systems is evolutionarily plastic [48], [49]. So far, evidence in *Nasonia* indicates that photoperiod changes are measured by a circadian timing system comprising a double oscillator [16]. Since in *Nasonia* also temperature influences diapause [50], differences in propensity to enter diapause may correlate with shifts in τ according to e.g. latitude. Differences in τ among *N. giraulti* strains tested in this study are virtually non-existent and may be explained by the fact that *N. giraulti* apparently contains very low genetic variation [20]. Although more variation is present among *N. longicornis* and *N. vitripennis* strains, no correlation is found between τ and latitude. The only correlation is seen between CoG in *N. vitripennis* females: strains from lower latitudes (Ita2 and Sal29, 45.7°N and 40.7°N, respectively) have a peak in activity earlier in the day than strains from higher latitudes (AsymC and HV3, 52.2°N). Despite finding consistent variation of circadian parameters (Tau, CoG) in populations of the cricket *D. fascipes* collected from 8°S to 43°N, Shimizu and Masaki [51] did not find any correlation with latitude. On the other hand, Joshi [43] found a correlation between circadian parameters in strains of *Drosophila ananassae* and the latitude at which these were collected, ranging from 6° to 34°N. Therefore, although our study did not aim directly at testing latitudinal clines in circadian parameters, results may depend on several factors like the species and the particular latitudinal range considered. However, since also induction of photoperiodism in *N. vitripennis* is known to correlate with latitude [45], more work is needed to understand whether also other circadian parameters follow a latitudinal gradient.

Other explanations for differences among strains are also plausible. Founder effects and rearing in the laboratory may influence circadian rhythms as discussed by others [52]. Additionally, since genes influencing circadian rhythms influence also other traits (e.g. developmental time, courtship behaviour) and rearing in the laboratory may affect these traits, a correlated effect of these causes on circadian rhythms is also expected.

## Conclusions

Our results confirm and extend previous findings to all four known *Nasonia* species. Remarkable differences are found in activity rhythms both among and within species and between sexes. Some differences, especially those among strains, may be caused by differences in geographical origin (i.e. different climatic conditions) or prolonged laboratory breeding. Differences among species that live in sympatry may contribute to temporal shifts in important life-history traits, such as mating or dispersal, and reduce the risk of interspecific hybridizations. Differences between sexes suggest that some sex-specific traits (e.g. emergence, mating) may be differently synchronized with circadian and annual rhythms. In conclusion, our results support the existence of chronotypic life-history differences among *Nasonia* species, strains and sexes but the functional significance of these differences requires further study.

## Supporting Information

Figure S1 **Examples of activity plots with rhythm splits.** These double-plots are examples in which activities have more than one internal period or display splits in the activity period. If the main period is sufficiently supported (see materials and methods) it is included in the analysis. *N. giraulti* RV2x(u) female with a main internal period (21.8 h) and a secondary period at roughly 25 h in LL (A). Two periods (23 and 26 h) are apparent in a *N. vitripennis* Sal29 female in LL (B). *N. longicornis* IV7R2 male with an unclear rhythm split in LL (C). Some *N. giraulti* VA1TET males show a 24 h-like rhythm in the first days in DD but then split. In this example, however, a main 22.6 h period can be seen from day 6 to 13 (D). ‘Wandering’ rhythms in a *N. vitripennis* HV3 male in DD: 22.5 h from day 6 to 10; 26 h from day 10 to 13 (E). Red dashed lines highlight multiple rhythms.
(TIF)

Figure S2 **Examples of activity plots with increasing ratio-to-p values.** In order to use a quantitative estimate to distinguish rhythmic vs. non-rhythmic activity plots, the ‘ratio-to-p’ value – the main peak value of the Qp statistics for rhythmicity (over the τ period tested) divided by the Qp value corresponding to the 0.01 probability threshold – was used as cut-off value. Activity plots with a ratio-to-p equal or lower than 5.5 were considered non-rhythmic. Here, examples of activity plots with increasing ratio-to-p values are shown. The values in the inset correspond to τ (in hours) and the ratio-to-p value, respectively, for the LL phase.
(TIF)

Figure S3 **Average activity in LD for additional** ***Nasonia*** **strains.** The median (with lower and upper quartiles) of the activity over the last three days in LD (double plotted) is indicated for each sex in additional *Nasonia* strains used for each species. Gray background indicates darkness. Y-axis: activity (pixel changes/minute); x-axis: hour of the day.
(TIF)

Figure S4 **Representative activity rhythms for** ***Nasonia*** **species.** Representative actograms to distinguish among *Nasonia* species and sexes are shown. For a description of activity patterns see Table S1. Additional actograms can be found in figures 1 and 7.
(TIF)

Table S1 **Classification of** ***Nasonia*** **species according to activity patterns and parameters in different light conditions.**
(TIF)
