# Supplementary figures and images for: Circadian Rhythms Differ between Sexes and Closely Related Species of Nasonia Wasps
Source: PLoS One. 2013 Mar 26;8(3):e60167. doi: 10.1371/journal.pone.0060167 (PMC3608630; doi:10.1371/journal.pone.0060167)

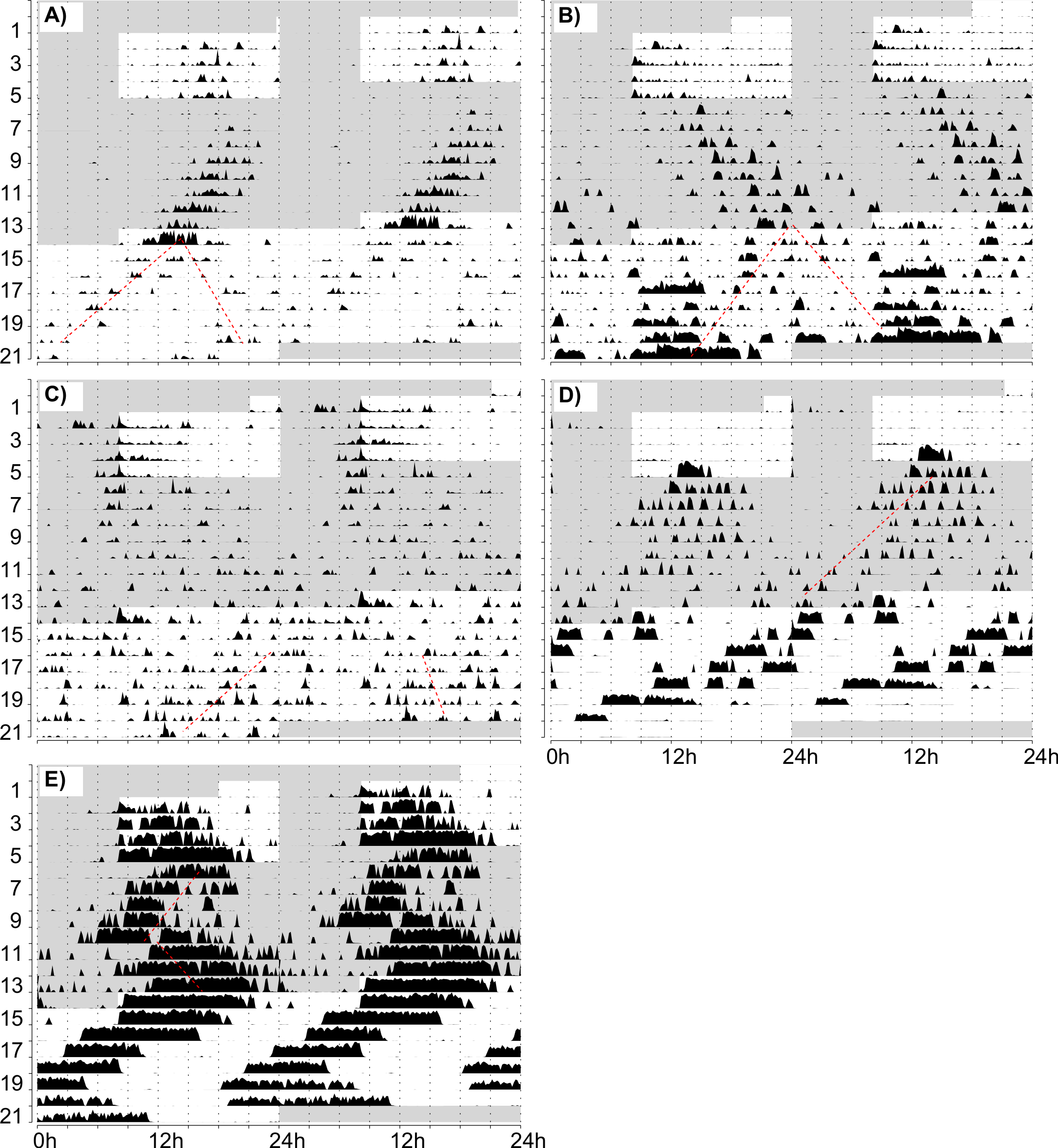

Supplement: Figure S1 — Examples of activity plots with rhythm splits. These double-plots are examples in which activities have more than one internal period or display splits in the activity period. If the main period is sufficiently supported (see materials and methods) it is included in the analysis. N. giraulti RV2x(u) female with a main internal period (21.8 h) and a secondary period at roughly 25 h in LL (A). Two periods (23 and 26 h) are apparent in a N. vitripennis Sal29 female in LL (B). N. longicornis IV7R2 male with an unclear rhythm split in LL (C). Some N. giraulti VA1TET males show a 24 h-like rhythm in the first days in DD but then split. In this example, however, a main 22.6 h period can be seen from day 6 to 13 (D). ‘Wandering’ rhythms in a N. vitripennis HV3 male in DD: 22.5 h from day 6 to 10; 26 h from day 10 to 13 (E). Red dashed lines highlight multiple rhythms. (TIF) [file pone.0060167.s001.tif]

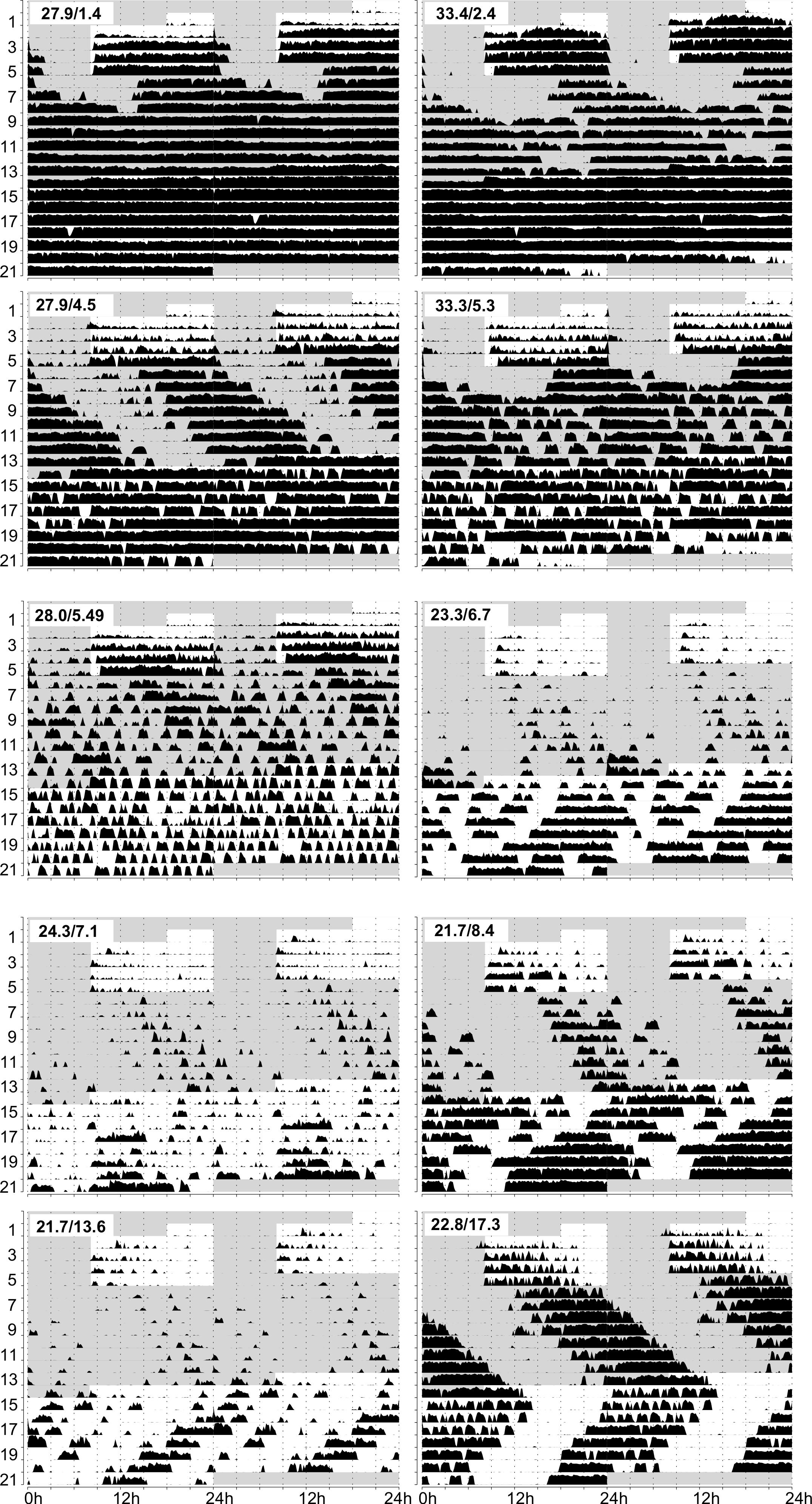

Supplement: Figure S2 — Examples of activity plots with increasing ratio-to-p values. In order to use a quantitative estimate to distinguish rhythmic vs. non-rhythmic activity plots, the ‘ratio-to-p’ value – the main peak value of the Qp statistics for rhythmicity (over the τ period tested) divided by the Qp value corresponding to the 0.01 probability threshold – was used as cut-off value. Activity plots with a ratio-to-p equal or lower than 5.5 were considered non-rhythmic. Here, examples of activity plots with increasing ratio-to-p values are shown. The values in the inset correspond to τ (in hours) and the ratio-to-p value, respectively, for the LL phase. (TIF) [file pone.0060167.s002.tif]

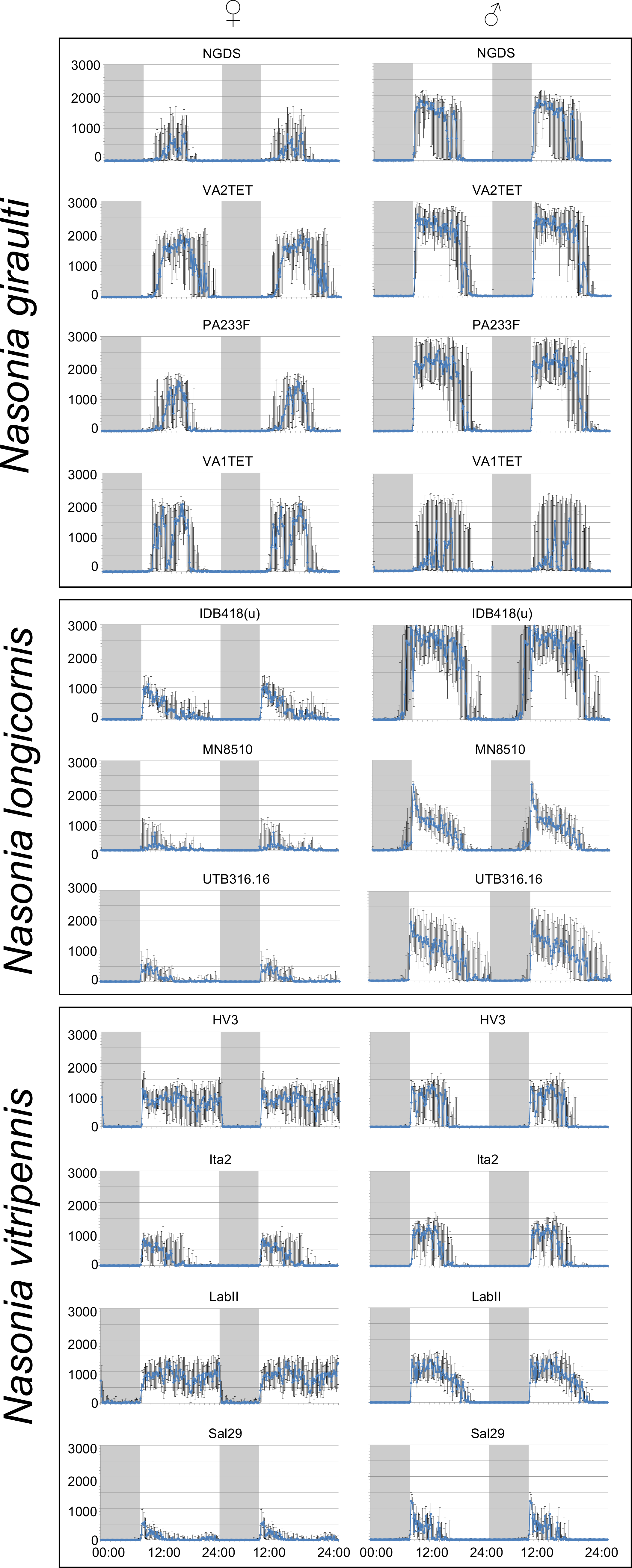

Supplement: Figure S3 — Average activity in LD for additional Nasonia strains. The median (with lower and upper quartiles) of the activity over the last three days in LD (double plotted) is indicated for each sex in additional Nasonia strains used for each species. Gray background indicates darkness. Y-axis: activity (pixel changes/minute); x-axis: hour of the day. (TIF) [file pone.0060167.s003.tif]

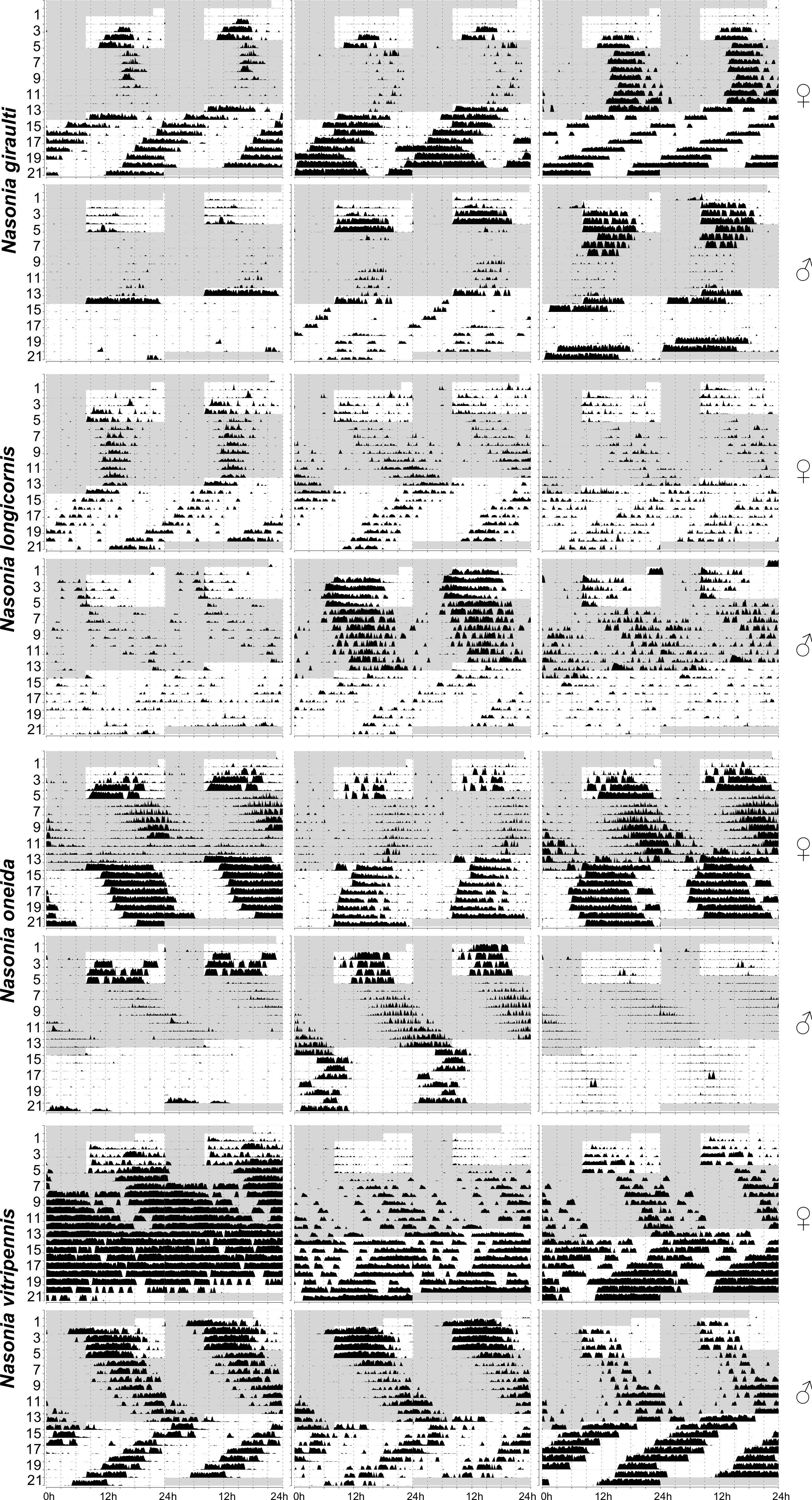

Supplement: Figure S4 — Representative activity rhythms for Nasonia species. Representative actograms to distinguish among Nasonia species and sexes are shown. For a description of activity patterns see Table S1. Additional actograms can be found in figures 1 and 7. (TIF) [file pone.0060167.s004.tif]

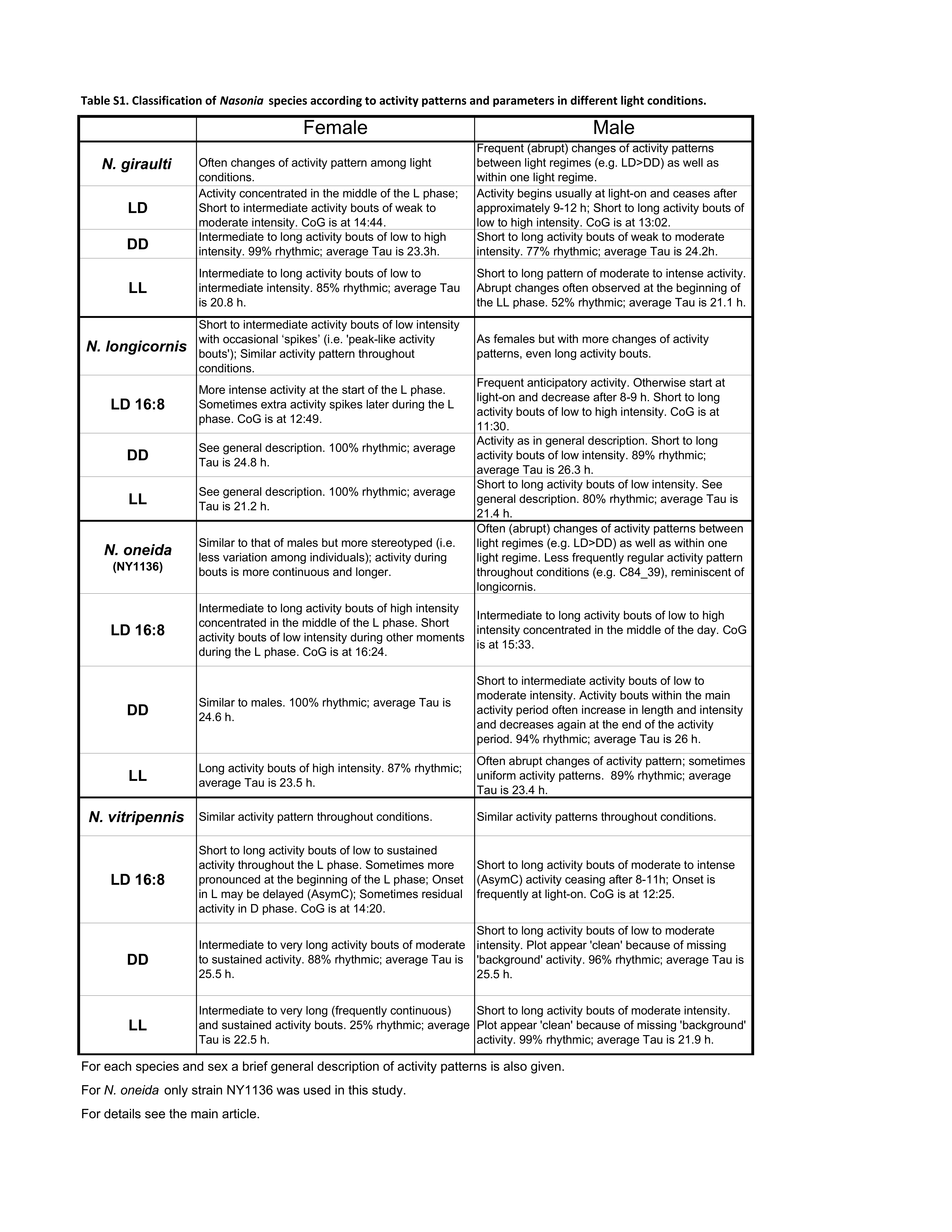

Supplement: Table S1 — Classification of Nasonia species according to activity patterns and parameters in different light conditions. (TIF) [file pone.0060167.s005.tif]
